# Inverse association of daily fermented soybean paste (“Jang”) intake with metabolic syndrome risk, especially body fat and hypertension, in men of a large hospital-based cohort

**DOI:** 10.3389/fnut.2023.1122945

**Published:** 2023-03-13

**Authors:** Su-Ji Jeong, Hee-Jong Yang, Hee Gun Yang, Myeong Seon Ryu, Gwangsu Ha, Do Yeon Jeong, Sunmin Park

**Affiliations:** ^1^Department of R&D, Sunchang Research Center for Fermentation Microbes, Sunchang-Gun, Republic of Korea; ^2^Department of Food and Nutrition, Obesity/Diabetes Research Center, Hoseo University, Asan, Republic of Korea; ^3^Department of Bioconvergence, Hoseo University, Asan, Republic of Korea

**Keywords:** fermented soybean paste, Jang, sodium, metabolic syndrome, body fat, hypertension

## Abstract

**Introduction:**

Jang is a fermented soybean paste containing salt and is traditionally used as a substitute for salt to enhance the flavor of foods in Korea. It has been speculated that regular consumption of Jang may lower the risk of metabolic syndrome (MetS). We hypothesized that Jang intake was associated with the risk of MetS and its components after adjusting for potential confounders, including sodium intake. The hypothesis was investigated according to gender in a large city hospital-based cohort (*n* = 58,701) in Korea.

**Methods:**

Jang intake, calculated as the sum of the intakes of Chungkookjang, Doenjang, Doenjang soup, and Ssamjang (a mixture of Doenjang and Kochujang), was included in the semi-quantitative food frequency questionnaire (SQFFQ) administered to the cohort, and the daily Jang intake was estimated. The participants were categorized into low-Jang and high-Jang groups by 1.9 g daily Jang intake. MetS was defined according to 2005 revised United States National Cholesterol Education Program-Adult Treatment Panel III (NCEP-ATP III) criteria modified for Asians.

**Results:**

The participants in the low-Jang and high-Jang groups consumed an average of 0.63 g and 4.63 g Jang daily; their total sodium intake was about 1.91 and 2.58 g/day, respectively. The participants in the high-Jang group had higher energy, fiber, calcium, vitamin C, vitamin D, and potassium intake than those in the low-Jang group. After adjusting for covariates, the participants with the highest sodium intake (≥3.31 g/day) were positively associated with MetS risk in the quintiles of men and women. Among the MetS components, waist circumference, fat mass, and hypo-high-density lipoprotein (HDL)-cholesterolemia were positively associated with sodium intake in all participants and women. Unlike the association seen with sodium intake, Jang intake (≥1.9 g/day) was inversely associated with MetS components, including waist circumference, fat mass, serum glucose concentrations, and hypo-HDL-cholesterolemia in all participants and men, after adjusting for covariates including sodium intake.

**Discussion:**

Substituting salt for Jang in cooking may be recommended to prevent and alleviate MetS incidence, and its efficacy for MetS risk was better in men than women. The results can be applied to sodium intake in Asian countries where salt is used to promote flavor.

## Introduction

From the olden days, Asians have consumed grains, mainly rice, as a staple food and soybeans and vegetables as side dishes. Koreans have traditionally used oils made by squeezing sesame and perilla seeds, which have been added to the side dishes to provide flavor (1). The salty taste was the primary flavor for foods, and salt also served as a preservative during the unavailability of the refrigerator (1). However, Koreans have recently substituted salt with various fermented soybeans or Jangs containing salts to promote flavor (2). Given the increasing evidence suggesting the role of excess sodium consumption as a risk factor for metabolic diseases, specifically hypertension, the health benefits of Jang intake have remained controversial (3, 4).

Soybeans were cultivated from 1,000–400 BC in Manchuria and from the Bronze Age in Korea (5). Seventy percent of Korea’s land is mountainous, and it is not suitable to raise animals for food. Therefore, white rice is the staple diet, and soybeans are an excellent complementary source of specific amino acids. They are cooked with rice and included in various side dishes. Like grains, soybeans can be dried for preservation but need to be soaked for over 12 h and boiled for 2–3 h before cooking. Soaking soybeans in water was difficult for every meal during winter in the olden days; hence, they were fermented with or without salts, making them easier to cook (5). The fermented soybeans include Doenjang, Chungkookjang, Kochujang, Kanjang, and SsamJang. Chungkookjang is the name given to soybeans fermented with rice straw but without salt at 42°C for two-three days, with salt added after fermentation for preservation (5). After fermentation, chungkookjang was also added with salt. Doenjang, Kochujang, and Kanjang are made from Meju, a dried and fermented brick of soybean without salt, for 50–60 days under cool and dry conditions. Meju is mixed with salt and water, fermented for over 6 months, and then the water component is separated (5). This water component is boiled and aged for over 6 months to form Kanjang, and the residues become Doenjang. Kochujang is produced by fermenting a mixture of Meju, glutinous rice powder, salts, and red pepper for over 6 months (6). Ssamjang is made of mixing Doenjang and Kochujang. Jang, a fermented soybean paste containing salt, is high in sodium (Doenjang 4.43 g/100 g; Ssamjang 3.01 g/100 g; Kochujang 2.40 g/100 g), and it has been used for providing salty and umami taste to various Hansik dishes substituting for salt in Korea.

Hansik is a Korean-style meal containing cooked multigrain rice, black soybean, soup, fish, two vegetable dishes, and kimchi (1). Jang is substituted for salt in all dishes, especially soup and vegetables, giving most dishes a better flavor (5). Koreans have consumed more sodium, probably due to salt-based meals for a long time. Significant data suggest that high salt intake increases the risk of hypertension, cardiovascular diseases, kidney diseases, and even gastric cancer (7). The World Health Organization (WHO) recommends the consumption of less than 2 g sodium intake (5 g salt) daily and the Dietary Approaches to Stop Hypertension (DASH) diet intervention for maintaining better metabolic health (7). In the Health Examinees (HEXA) Study, a large-scale genomic cohort study, Korean adults in the highest quartile of sodium intake were at a higher risk of developing the metabolic syndrome (MetS) (OR = 1.11) than those in the lowest quartiles after adjusting for covariates (8). Furthermore, potassium intake was found to be inversely associated with hypertension risk according to the data from the Korea National Health and Nutrition Examination Survey (KNHANES) 2007–2012 (9). Sodium intake is higher in adults on a high-Hansik diet than those on a low-Hansik diet, yet a high-Hansik diet is inversely associated with MetS risk and diastolic blood pressure (10). Gender differences are also seen when examining the effect of diets on MetS risk (11, 12). Although a high Hansik intake has high sodium, it is suggested to improve hypertension and MetS risk. Therefore, the relationship between salt and the development of metabolic diseases may not be related to the amount of salt consumed *per se* but rather to what kind of foods containing salt is consumed.

Previous human and animal studies have shown that consuming Chungkookjang, Doenjang, Kanjang, and Kochujang lowers weight gain and improves insulin resistance, dyslipidemia, and hyperglycemia (6, 13–17). Jang is used as a substitute for table salt and could be one of the potential candidates to reduce MetS risk. However, no large epidemiologic study has been conducted to date on the effect of Jang intake on MetS. We hypothesized that Jang intake, including Chungkookjang, Doenjang, and Ssamjang, was associated with MetS risk after adjusting for potential confounders, including sodium intake. The hypothesis was investigated in subjects of both genders in a large city hospital-based cohort (*n* = 58,701) in Korea.

## Methods

### Participants

A total of 58,701 volunteers (20,293 men and 38,408 women) were recruited from an urban hospital-based cohort involving multi-institutional hospitals in major cities in Korea. This cohort was part of the Korean Genome and Epidemiology Study (KoGES) conducted by the Korean National Research Institute of Health (NIH), the Korea Disease Control and Prevention Agency (KDCA), and the Ministry of Health and Welfare (MOHW) of Korea. The cohort was made aware of the public health issues associated with metabolic diseases (18). The inclusion criteria for recruiting participants were age ≥40 and the presence of mild to moderate metabolic disease states at baseline (18). The study was conducted after ethical approval from the Institutional Review Board of the National Institute of Health, Korea (KBP-2015-055) and Hoseo University, Korea (HR-034-01). The participants signed written informed consents.

### Anthropometric and biochemical measurements

On their visits to the hospital, the participants wore light clothes and removed their shoes to measure height, weight, waist, and hip circumference (19). Body mass index (BMI) was determined by dividing the body weight (kg) by height in m squared (m^2^). The waist circumference was measured at the midpoint between the lower border of the rib cage and the iliac crest, at the level of the umbilicus, using a flexible inch tape. Body fat and skeletal muscle mass were estimated using a machine-learning prediction model generated in the Ansan/Ansung cohort. These parameters were measured using the Inbody 3.0 measurement device (Cheonan, Korea) based on the principle of bioelectrical impedance (20). Skeletal muscle index (SMI) was calculated by dividing the appendicular skeletal muscle mass by height. A doctor measured blood pressure in the left arm in a sitting position with a sphygmomanometer.

After the subjects undertook an overnight fast, their blood was drawn using vacuum blood collection tubes with and without ethylenediaminetetraacetic acid (EDTA). Separated plasma and serum samples were used for biochemical analysis. Glucose, total cholesterol, high-density lipoprotein (HDL) cholesterol, triglycerides, creatinine concentrations, and aspartate aminotransferase (AST) and alanine aminotransferase (ALT) activities were assayed from the fasting serum or plasma samples using a Hitachi 7,600 automatic analyzer (Hitachi, Tokyo, Japan). Glycated hemoglobin or hemoglobin A1c (HbA1c) was determined using an HbA1c Analyzer from EKF Diagnostics (Manchester, United Kingdom). In the Ansan/Ansung cohort, insulin resistance was also estimated using the prediction model generated by the homeostatic model assessment for insulin resistance (HOMA-IR) equation. The HOMA-IR is calculated by multiplying fasting serum glucose concentrations (mg/dL) by insulin (mU/L) concentrations and dividing the result by 405 (21). The participants were classified into low-and high-insulin resistance with the cutoff of 2.32 HOMA-IR value (21). The serum low-density lipoprotein (LDL) cholesterol concentrations were calculated using the Friedewald equation, and subjects with serum triglyceride concentrations ≥500 mg/dl were excluded. Serum high-sensitive C-reactive protein (hs-CRP) concentrations were measured with an enzyme-linked immunoassay (ELISA) kit (R&D Systems, Minneapolis, MN, United States). The estimated glomerular filtration rate (eGFR) was calculated using the modification of diet in renal disease (MDRD) formula: 175 X serum creatinine concentration^−1.154^ X age^−0.203^ X [0.742 if female].

Physical exercise was determined based on the questionnaires about exercise intensity and duration, and the intensity was categorized into light, moderate, and intensive. The light exercise included walking, office work, and dishwashing; moderate exercise included brisk walking, mowing the lawn, badminton, swimming, and tennis; and intensive exercise included climbing, running, football, basketball, and volleyball. Regular physical exercise was defined as daily exercise with a weekly aggregate of over 150 min of moderate exercise or over 100 min of intense exercise. The participants who did not belong to the regular or intense exercise groups were considered part of the low-exercise group. Alcohol consumption was assessed based on the type, amount, and frequency of alcohol intake during the previous 6 months. Daily alcohol intake was calculated by multiplying the drinking frequency by the average alcohol consumed on each occasion and dividing it by the period in days. Smoking status was stratified into current, past, or never based on >100 cigarettes smoked over the lifetime and smoking during the last 6 months before joining the study.

### MetS definition

MetS was defined based on 2005 revised National Cholesterol Education Program-Adult Treatment Panel III (NCEP-ATP III) criteria for Asians. The cutoff of waist circumference was ≥90 cm for men and >85 cm for women, as established by the Korean Society for the Study of Obesity (KSSO), since they increase MetS risk in lower waist circumferences than Caucasians (22, 23). The persons taking medication for hyperglycemia, dyslipidemia, and hypertension were assigned to the MetS group, although they had serum glucose concentration, lipid profiles, and blood pressure within a normal range.

### Daily food and nutrient intake

The KoGES committee established a semi-quantitative food frequency questionnaire (SQFFQ) for Koreans, including 106 foods commonly consumed in meals (24). The SQFFQ was validated from the 3-day food records in four seasons (25). The SQFFQ was filled by the participants based on food intake for the last 6 months and included the consumption frequency and amounts of the 106 food items with the selected serving sizes. The results of the food intake from the SQFFQ were converted into the intake of 23 nutrients, including sodium, using the computer-aided nutritional analysis program 3.0 developed by the Korean Nutrition Society (24). The nutrient contents of the processed and cooked foods in SQFFQ were calculated based on the typical recipes. Sodium intake was calculated from the food intake determined from SQFFQ (8). Lee et al. (8) calculated sodium intake using the SQFFQ, the same one used in the present study. The salt contents of the foods, such as Kimchi, Doenjang, and soup, were estimated using their regular recipes, although condiment contents, including salt, can be varied according to the individuals.

### Jang and sodium intake

Jang, a fermented soybean paste containing salt, was included as a food group in the SQFFQ, and it included Doenjang soup, Doenjang, Chungkookjang, and Ssamjang. The daily Jang intake was calculated from the frequencies and portion sizes of these four items as the other food items. Sodium intake from the Jang intake was calculated using the computer-aided nutritional analysis program 3.0. Due to different variations of Jang and sodium intake, Jang intake was categorized into tertiles or two groups (cutoff: 1.9 g/day; 25th percentile), while sodium intake was stratified into quintiles and two groups (cutoff: 1.5 g/day; 25th percentile).

### Dietary pattern analysis and dietary inflammatory index

The 106 food items were classified into 29 pre-combined food groups and used as independent variables in a principal component analysis (PCA) conducted to find the optimal factors representing dietary patterns in Korea. The optimal number of the classified factors was estimated based on eigenvalues of >1.5 in the PCA. In this cohort, the number of factors that met the criteria was four. The orthogonal rotation procedure (Varimax) was applied to generate the appropriate clusters, indicating dietary patterns (26). Dietary factor-loading values of ≥0.40 were used to indicate significant contributions of food items by assigning names to the dietary patterns (26). The primary food groups in the four different dietary patterns were fish, crabs, red meat, vegetables, kimchi, pickles, seaweed, and mushrooms for the Korean balanced diet (KBD); noodles, bread, fast foods, soups, and meat for the Western-style diet (WSD); beans, potatoes, green vegetables, seaweed, milk, nuts, and eggs for the plant-based diet (PBD); and rice in the rice-main diet (RMD).

The dietary inflammatory index (DII) was calculated based on the dietary inflammatory weights of foods and nutrients having anti-or proinflammatory properties (energy, 32 nutrients, four food products, four spices, and caffeine). The inflammatory weights of the foods and nutrients in the DII equation were adopted from a previous study (27). However, the intake of the four spices, garlic, ginger, saffron, and turmeric, was removed from the DII original equation since they were not measured in the SQFFQ. The DII was calculated by multiplying the pro-(plus value) and anti-inflammatory weights (minus value) of the 38 dietary components by their daily intake and then dividing the sum of each item by 100.

### Stress status

Stress status was evaluated with 18 questions about physical and psychological stress at home and work, and each question was scored from zero (lowest stress) to three (highest stress). The overall stress status scores were estimated by a summation of the scores of the 18 questions, and higher stress status scores indicated that the participant’s stress status was high.

### Statistical analyses

Statistical analyses were carried out using the SAS version 9.3 software (SAS Institute, Cary, NC, United States). When the sample size was calculated using the G*Power program with effect size (0.05), significant level (α = 0.05), and power (β = 0.99), the sample size derived was 1,036. The sample size was satisfied for each gender. The descriptive statistics for the categorical variables (e.g., gender and lifestyle) were evaluated based on the frequency distributions of low-and high-Jang intake in each gender. The statistical differences in the frequency distributions were measured using the Chi-squared test. Adjusted means and standard errors of the low-Jang and high-Jang intake groups in each gender were calculated for continuous variables. The statistical differences between low-and high-Jang intake were determined using the analysis of covariance (ANCOVA) after adjusting for covariates, including age, residence area, education, income, energy intake, sodium intake, alcohol consumption, physical exercise, and smoking status. The adjusted odds ratio (ORs) and 95% confidence intervals (CI) of Jang intake with MetS risk were measured by multiple regression analysis after covariate adjustment. *p* values <0.05 were considered to be statistically significant.

## Results

### Baseline characteristics of the participants

There was no difference in age between the low-and high-Jang intake groups. The participants, both men and women, with below high school education and income below 4,000 dollars per month, had a lower Jang intake than those with over high school education and income over 4,000 dollars (Table 1). Participants who exercised regularly consumed more Jang than those with a sedentary lifestyle. An evaluation of Jang intake based on the smoking status showed that male non-smokers had a lower Jang intake than former and current smokers, but women showed the opposite trend (Table 1). Alcohol intake was higher in men than women but did not vary with the Jang intake in both men and women. The stress status was linked to Jang intake, and both men and women participants with a high Jang intake had lower stress scores.

**Table 1:** General characteristics according to gender and Jang intake.

	Men (*n* = 20,293)		Women (*n* = 38,408)	
	Low-Jang (*n* = 4,467)	High-Jang (*n* = 15,826)	Low-Jang (*n* = 8,646)	High-Jang (*n* = 29,762)
Age (years)	57.0 ± 0.13^a^	56.9 ± 0.10^a^	52.5 ± 0.10^b^	52.4 ± 0.07^b***^
*Education*				
≤Middle school	324 (12.3)	1,429 (14.5)	1,249 (19.1)	5,489 (23.0)
High school	2008 (76.4)	7,426 (75.4)	4,839 (73.9)	17,032 (71.4)
≥College	296 (11.3)	995 (10.1)^⁑⁑^	457 (6.98)	1,325 (5.56)^⁑⁑⁑^
*Income*				
≤$2000	327 (7.69)	1,279 (8.49)	955 (11.7)	3,217 (11.5)
$2000–4,000	1,727 (40.6)	6,480 (43.0)	3,391 (41.7)	12,594 (45.0)
>$4,000	2,201 (51.7)	7,299 (48.5)^⁑⁑^	3,792 (46.6)	12,178 (43.5)^⁑⁑⁑^
Physical exercise (%)	2,534 (57.2)	9,418 (59.6)^⁑⁑^	4,356 (50.8)	15,668 (52.8)^⁑⁑^
Former smoking	1834 (41.3)	6,961 (44.1)	116 (1.35)	344 (1.16)
Smoking (%)	1,212 (27.9)	4,452 (28.2)^⁑⁑⁑^	195 (2.27)	554 (1.87)^⁑^
Alcohol (g/day)	30.8 ± 1.16^a^	30.7 ± 0.69^a^	9.01 ± 0.86^b^	9.02 ± 0.48^b***^
Stress score (scores)	14.0 ± 0.16^c^	13.2 ± 0.09^d^	16.1 ± 0.12^a^	15.2 ± 0.07^b***+++^

Values represents means and standard deviations for continuous variables and numbers and percentage for categorical variables. The cutoffs of Jang intake were <1.9 g/day. Stress status was estimated with 18 questions about the physical and psychological at home and work, and each question was scored 0 (lowest stress) to 3 (highest stress). The scores of the stress status were estimated with a summation of the scores of 14 questions. The higher scores of the stress status indicated having higher stress. ^***^Significant differences by genders at *p* < 0.001. ^+++^Significant differences by Jang intake at *p* < 0.001. ^‡‡‡^Significantly different from the low-Jang group in *χ*^2^ test in each gender at *p* < 0.001. ^a,b,c,d^Different superscripts on the values indicated significant differences among the groups in Tukey’s test at *p* < 0.05.

### Food intake according to gender in the Jang intake groups

The men and women participants with a high Jang intake consumed 4.62 ± 0.03 and 4.64 ± 0.02 g Jang per day, respectively, while those with a low Jang intake consumed 0.60 ± 0.05 and 0.65 ± 0.04 g/day, respectively (Table 2). The multigrain rice intake was higher, but white rice intake was lower in male participants with a high Jang intake compared to those with a low Jang intake. Noodle and bread intakes were also higher in the low-Jang intake group than in the high-Jang intake group for both genders (Table 2). The intake of fruits and vegetables was higher in the high-Jang intake group than in the low-Jang intake group. Furthermore, the intake of kimchi, a fermented cabbage, was much higher in the high-Jang intake group than in the low-Jang intake group for both genders (Table 2). The intakes of seaweed, fish, beans, and nuts were higher in the high-Jang intake group than in the low-Jang intake group, but meat intake showed a trend opposite to that of seaweed. There were twice as many participants with high Jang intake in the KBD and PBD compared to those with low Jang intake in both genders. However, the trend was reversed in the participants having the RMD (Table 2).

**Table 2:** Food intake according to gender and Jang intake.

	Men (*n* = 20,293)		Women (*n* = 38,408)	
	Low-Jang (*n* = 4,467)	High-Jang (*n* = 15,826)	Low-Jang (*n* = 8,646)	High-Jang (*n* = 29,762)
Jang (g/day)	0.92 ± 0.08^b^	4.72 ± 0.05^a^	0.87 ± 0.06^b^	4.60 ± 0.04^a+++^
Multigrain rice (g/day)	526 ± 5.57^b^	543 ± 3.32^a^	467 ± 4.14^c^	462 ± 2.39^c***###^
White rice (g/day)	138 ± 4.97^a^	117 ± 2.97^b^	63.3 ± 3.70^c^	65.1 ± 2.14^c***++###^
Noodles (g/day)	65.4 ± 1.60^b^	64.7 ± 0.96^a^	46.9 ± 1.19^c^	37.8 ± 0.69^d***+++###^
Bread (g/day)	14.2 ± 0.52^a^	12.9 ± 0.31^b^	15.5 ± 0.38^a^	13.0 ± 0.22^b***++^
Fruits (g/day)	194 ± 4.72^c^	210 ± 2.82^b^	243 ± 3.51^a^	249 ± 2.03^a***+++#^
Vegetables (g/day)	241 ± 3.64^c^	289 ± 2.18^a^	209 ± 2.71^d^	255 ± 1.57^b***+++###^
Kimchi (g/day)	135 ± 2.40^b^	163 ± 1.43^a^	108 ± 1.79^c^	131 ± 1.03^b***+++^
Seaweeds (g/day)	1.58 ± 0.05^c^	1.91 ± 0.03^b^	1.80 ± 0.03^b^	2.27 ± 0.02^a***+++##^
Fish (g/day)	39.8 ± 0.85^b^	43.8 ± 0.51^a^	36.8 ± 0.63^c^	39.0 ± 0.36^b***+++^
Meats (g/day)	45.5 ± 0.87^a^	43.6 ± 0.52^a^	32.1 ± 0.65^b^	27.7 ± 0.37^c***+++##^
Beans^1^ (g/day)	33.2 ± 1.19^b^	45.0 ± 0.71^a^	32.1 ± 0.88^b^	43.9 ± 0.51^a+++^
Nuts (g/day)	1.31 ± 0.10^c^	1.79 ± 0.06^b^	2.01 ± 0.07^b^	2.42 ± 0.04^a***++^
KBD (%)	982 (22.7)	6,489 (41.0)^⁑⁑⁑^	1,540 (17.8)	10,445 (35.1)^⁑^
PBD (%)	635 (14.2)	3,646 (23.0)^⁑⁑⁑^	2,194 (25.4)	11,572 (38.9)^⁑⁑⁑^
WSD (%)	2024 (45.3)	8,179 (51.7) ^⁑⁑⁑^	2,794 (32.3)	10,452 (35.1) ^⁑⁑⁑^
RMD (%)	1,657 (37.1)	5,100 (32.2) ^⁑⁑⁑^	3,177 (36.7)	9,387 (31.5)^⁑⁑⁑^

Values represents means and standard deviations for continuous variables and numbers and percentage for categorical variables. ^1^Beans without Jang intake. The cutoffs of Jang intake were <1.9 g/day. ^***^Significant differences by genders at *p* < 0.001. ^+++^Significant differences by Jang intake at *p* < 0.01 and ^+++^ at *p* < 0.001. ^#^Significant interaction between genders and Jang intake at *p* < 0.05, ^##^ at *p* < 0.01, ^###^*p* < 0.001. ^‡‡‡^Significantly different from the low-Jang group in *χ*^2^ test in each gender at *p* < 0.001. ^a,b,c^Different superscripts on the values indicated significant differences among the groups in Tukey’s test at *p* < 0.05.

### Nutrient intake according to gender in the Jang intake groups

Energy intake did not meet the estimated energy requirement (EER) in men regardless of their Jang intake. In women, the EER was met only in the participants in the high-Jang intake group (Table 3). Carbohydrate intake was lower in the high-Jang intake group compared to the low-Jang intake group in both genders. However, the trends of protein and fat intakes were opposite to that of the carbohydrate intake in both genders. Fiber and calcium intakes were 1.3-fold higher in the high-Jang intake group than in the low-Jang intake group in both genders (Table 3). Along with sodium, potassium intake was higher in the high-Jang intake than in the low-Jang intake groups in both genders. However, the potassium-to-sodium intake ratio was lower in the high-Jang intake group compared to the low-Jang intake group in both genders. Dietary consumption of vitamin C did not meet the recommended intake levels (100 mg/day) in the low-Jang intake group but was met in the high-Jang intake group (Table 3). Similarly, vitamin D consumption also did not reach the recommended intake levels, although it was much higher in the high-Jang intake group than in the low-Jang intake group. DII was lower in the high-Jang intake group than in the low-Jang intake group for both genders. Moreover, the intakes of total polyphenols, flavonoids, and isoflavonoids were higher in the high-Jang intake group than in the low-Jang intake group for both genders (Table 3). These results indicated that the participants with a high Jang intake consumed a high-quality diet compared to those with a low Jang intake.

**Table 3:** Nutrient intake according to gender and Jang intake.

	Men (*n* = 20,293)		Women (*n* = 38,408)	
	Low-Jang (*n* = 4,467)	High-Jang (*n* = 15,826)	Low-Jang (*n* = 8,646)	High-Jang (*n* = 29,762)
Energy (EEE%)	81.7 ± 0.67^d^	92.8 ± 0.41^c^	90.1 ± 0.50^b^	104 ± 0.29^a***+++##^
Carbohydrate (En%)	73.3 ± 0.15^a^	71.5 ± 0.09^b^	72.8 ± 0.11^a^	71.1 ± 0.07^c**+++^
Fat (En%)	12.7 ± 0.12^d^	13.9 ± 0.07^b^	13.2 ± 0.09^c^	14.3 ± 0.05^a***+++^
Protein (En%)	12.5 ± 0.06^d^	13.4 ± 0.03^b^	12.9 ± 0.04^c^	13.9 ± 0.02^a***+++^
Fiber (g/day)	12.2 ± 0.21^c^	16.2 ± 0.13^a^	11.5 ± 0.16^d^	15.4 ± 0.09^b***+++^
Calcium (mg/day)	339 ± 5.82^d^	453 ± 3.50^b^	383 ± 4.35^c^	509 ± 2.50^a*+++^
Magnesium (mg/day)	429 ± 2.25^b^	448 ± 1.66^a^	379 ± 1.67^d^	392 ± 1.19^c***+++^
Potassium (mg/day)	1,812 ± 23.3^c^	2,336 ± 14.1^a^	1,905 ± 17.5^c^	2,453 ± 10.0^b***+++^
Sodium from all foods (mg/day)	2,046 ± 20.6^c^	2,752 ± 10.7^a^	1,833 ± 14.9^d^	2,497 ± 7.83^b***+++^
Sodium from Jang (mg/day)	25.9 ± 1.99^b^	199 ± 1.29^a^	28.0 ± 1.72^b^	200 ± 0.86 ^a+++^
Ratio of *K* and Na	1.13 ± 0.01^b^	0.95 ± 0.006^d^	1.27 ± 0.007^a^	1.07 ± 0.004^c***+++^
Vitamin C (mg/day)	78.8 ± 1.53^d^	106 ± 0.792^b^	91.4 ± 1.14^c^	122 ± 0.66^a***+++###^
Vitamin D (ug/day)	4.84 ± 0.10^c^	6.27 ± 0.08^b^	6.02 ± 0.10^b^	7.66 ± 0.06^b+^
DII (scores)	−18.8 ± 1.83^ab^	−23.3 ± 1.19a	−14.7 ± 1.53^a^	−23.3 ± 0.96^b+++^
Total polyphenol (mg/day)	2,417 ± 24.7^b^	2,758 ± 14.9^a^	2,339 ± 18.5^b^	2,634 ± 10.6^c***+++^
Flavonoids (mg/day)	26.3 ± 0.72^d^	36.7 ± 0.44^b^	35.5 ± 0.54^c^	45.7 ± 0.31^a***+++^
Isoflavonoids (mg/day)	5.25 ± 0.17^d^	8.86 ± 0.10^b^	6.06 ± 0.12^c^	9.80 ± 0.07^a***+++^

Values represents means and standard deviations for continuous variables and numbers and percentage for categorical variables. The cutoffs of Jang intake were <1.9 g/day. EEE, estimated energy requirement. ^***^Significant differences by genders at *p* < 0.001. ^+++^Significant differences by Jang intake at *p* < 0.001. ^#^Significant interaction between genders and Jang intake at *p* < 0.05, ^##^ at *p* < 0.01, ^###^ *p* < 0.001. ^‡^Significantly different from the low-Jang group in *χ*^2^ test in each gender at *p* < 0.05, ^‡‡^ at *p* < 0.01, ^‡‡‡^ at *p* < 0.001. ^a,b,c,d^Different superscripts on the values indicated significant differences among the groups in Tukey’s test at *p* < 0.05.

### Association between sodium intake and the risk of MetS components

Waist circumferences and fat mass were higher in the high-sodium group than in the low-sodium group in both genders. Interestingly, SMI was also higher in the high-sodium group than the low-sodium group (Table 4). There were no significant differences in serum glucose, LDL, HDL, AST, and ALT concentrations, HbA1c levels, insulin resistance by HOMA-IR, and systolic blood pressure (SBP) between the two groups. Serum triglycerides and diastolic blood pressure (DBP) were slightly higher in the high-sodium group than in the low-sodium group (Table 4). eGFR was also higher in the high-sodium group than in the low-sodium group, indicating that excess sodium from the diet can be removed through urine excretion.

**Table 4:** Adjusted means of metabolic parameters according to gender and daily sodium intake and their associations.

	Men (*n* = 20,293)	Women (*n* = 38,408)		
	Low-Jang (*n* = 4,467)	High-Jang (*n* = 15,826)	Low-Jang (*n* = 8,646)	High-Jang (*n* = 29,762)
BMI (mg/kg^2^)	24.5 ± 0.05^a^	24.6 ± 0.04^a^	23.4 ± 0.04^c^	23.5 ± 0.03^b***++^
Waist circumferences (cm)	85.6 ± 0.14^a^	85.9 ± 0.11^a^	77.1 ± 0.11^c^	77.6 ± 0.08^b***+++^
SMI (kg/m)	7.19 ± 0.01^b^	7.25 ± 0.01^a^	6.04 ± 0.01^d^	6.10 ± 0.01^c***+++^
Fat mass (%)	23.3 ± 0.07^c^	23.4 ± 0.05^c^	30.3 ± 0.05^b^	30.5 ± 0.04^a***++^
Serum glucose (mg/dL)	99.9 ± 0.35^a^	98.9 ± 0.28^a^	93.3 ± 0.24^b^	92.9 ± 0.22^b***^
HbA1c (%)	5.79 ± 0.02^a^	5.77 ± 0.01^a^	5.68 ± 0.01^b^	5.67 ± 0.01^b***^
Insulin resistance (*N*, %)	667 (11.1)	1,645 (11.5)	727 (6.16)	1,565 (5.88)
Serum total cholesterol (mg/dL)	191 ± 0.68^b^	191 ± 0.50^b^	200 ± 0.51^a^	200 ± 0.36^a***^
Serum HDL (mg/dL)	49.5 ± 0.25^b^	49.3 ± 0.18^b^	57.5 ± 0.18^a^	57.1 ± 0.13^a***^
Serum LDL (mg/dL)	115 ± 0.63^b^	115 ± 0.46^b^	120 ± 0.47^a^	120 ± 0.33^a***^
Serum triglyceride (mg/dL)	132 ± 1.55^a^	136 ± 1.13^a^	114 ± 1.16^b^	115 ± 0.82^b***+^
SBP (mmHg)	127 ± 0.26^a^	127 ± 0.21^a^	120 ± 0.18^b^	120 ± 0.17^b***^
DBP (mmHg)	78.5 ± 0.18^a^	79.0 ± 0.13^a^	73.8 ± 0.14^b^	74.1 ± 0.10^b***++^
Serum hs-CRP (mg/dL)	0.17 ± 0.01^a^	0.15 ± 0.01^a^	0.12 ± 0.01^b^	0.13 ± 0.01^b***#^
eGFR (ml/min/1.73 m^2^)	81.7 ± 0.29^d^	82.1 ± 0.22^c^	87.5 ± 0.19^b^	88.0 ± 0.16^a***++^
Serum AST (U/L)	25.0 ± 0.22^a^	25.4 ± 0.16^a^	22.9 ± 0.16^b^	22.7 ± 0.12^b***#^
Serum ALT (U/L)	25.2 ± 0.33^a^	25.8 ± 0.24^a^	20.2 ± 0.24^b^	19.9 ± 0.17^b***^

Values represent means and standard deviations for continuous variables and numbers and percentages for categorical variables. Covariates for adjustment included age, energy intake, residence area, education, income, sodium and alcohol intake, smoking status, and physical activity. The cutoff of sodium intake was <1.5 g/day. BMI, body mass index, SMI, skeletal muscle index; HbA1c, hemoglobin A1c; HOMA-IR, Homeostatic Model Assessment for Insulin Resistance (cutoff: 2.32 of HOMA-IR); HDL, high-density lipoprotein; LDL, low-density lipoprotein; SBP, systolic blood pressure; DBP, diastolic blood pressure; hs-CRP, high-sensitive C-reactive protein; AST, aspartate aminotransferase; ALT, alanine aminotransferase. ^‡^Significantly different from the low-Jang group in *χ*^2^ test in each gender at *p* < 0.05. ^***^Significant differences by genders at *p* < 0.001. ^+^Significant differences by sodium intake at *p* < 0.01, ^++^*p* < 0.01 and ^+++^ at *p* < 0.001. ^#^Significant interaction between genders and sodium intake at *p* < 0.05. ^a,b,c,d^Different superscripts on the values indicated significant differences among the groups in Tukey’s test at *p* < 0.05.

**Figure 1:**
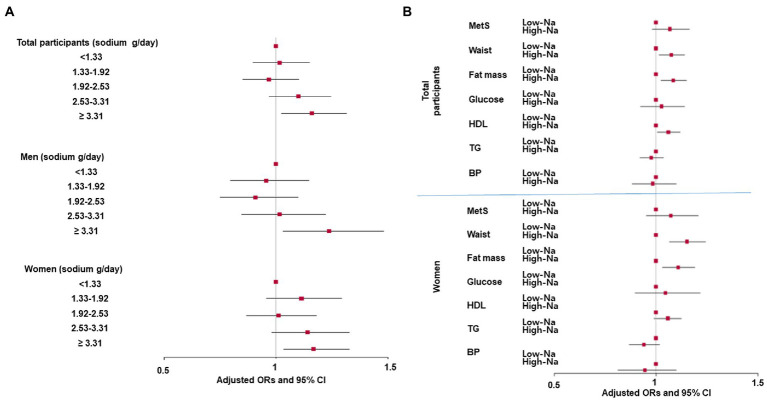
Association of sodium intake with metabolic syndrome (MetS) and its components according to gender. **(A)** Adjusted odds ratio and 95% confidence intervals of MetS with sodium intake by quintiles and **(B)** Adjusted odds ratio and 95% confidence intervals of MetS and its components with low-and high-sodium intake. (Cutoff: 1.5 g sodium intake/day) The cutoff point of each MetS component for logistic regression were as follows: MetS criteria based on the 2005 NCEP-ATP III criteria for Asians; <90 cm for men and 85 cm for women waist circumferences; <25% for men and 30% for women for fat mass; <110 mg/dL fasting serum glucose plus diabetic drug intake; <40 mg/dL for men and 50 mg/dL for women serum HDL cholesterol; <140 mmHg systolic blood pressure (SBP) or diastolic blood pressure (DBP) <90 mmHg plus hypertension medication.

The highest sodium intake (≥3.31 g/day) was positively associated with MetS risk by 1.3 times based on the lowest sodium intake (<1.33 g/day) in the quintile categories of all participants (Figure 1A). The association was not shown in gender difference (Figure 1A). However, sodium intake was not associated with MetS risk in all the participants when daily sodium intake was divided into two groups by 1.5 g/day. The cutoff was assigned conservatively, less than WHO recommended sodium intake (2 g sodium/day; 5 g NaCL/day) since the sodium intake measured from the SQFFQ could be underestimated. Sodium intake was associated with some components of MetS in all participants: A positive association with waist circumference and fat mass and an inverse association with serum HDL concentrations were observed with sodium intake. However, there was no association of sodium intake with serum glucose and triglyceride concentrations and blood pressure in all participants (Figure 1B). There was no association between sodium intake and the risk of MetS and its components in men. However, sodium intake was positively associated only with waist circumferences and fat mass only in women (Figure 1B).

### Association between Jang intake and the risk of MetS components

BMI, waist circumference, SMI, fat mass, serum glucose concentrations, HbA1c levels, and insulin resistance were not significantly different between the low-and high-Jang intake groups in both genders (Table 5). In the lipid profiles, the serum triglyceride concentrations showed an interaction with gender types, but there was no significant difference between the low-and high-Jang groups in both genders (Table 5). There were no significant differences in the serum total cholesterol, HDL, and LDL concentrations in the low-and high-Jang intake groups. Serum hs-CRP concentration, an inflammation index, was lower in the high-Jang intake group than in the low-Jang intake group. SBP, DBP, eGFR, and serum AST and ALT concentrations were not significantly different between the low-and high-Jang intake groups, although they showed a gender difference (Table 5).

**Table 5:** Adjusted means of metabolic parameters according to gender and Jang intake and their associations.

	Men (*n* = 20,293)	Women (*n* = 38,408)		
	Low-Jang (*n* = 4,467)	High-Jang (*n* = 15,826)	Low-Jang (*n* = 8,646)	High-Jang (*n* = 29,762)
BMI (mg/kg^2^)	24.7 ± 0.06^a^	24.5 ± 0.04^b^	23.5 ± 0.04^c^	23.4 ± 0.03^c***+^
Waist (cm)	85.6 ± 0.17^a^	85.3 ± 0.11^a^	77.5 ± 0.13^c^	77.8 ± 0.07^b***#^
SMI (kg/m)	7.27 ± 0.02^a^	7.27 ± 0.01^a^	6.07 ± 0.01^b^	6.06 ± 0.01^b***^
Fat mass (%)	23.4 ± 0.08^b^	23.3 ± 0.05^b^	30.4 ± 0.06^a^	30.4 ± 0.03^a***^
Serum glucose (mg/dL)	99.5 ± 0.38^a^	99.1 ± 0.28^a^	93.4 ± 0.28^b^	93.0 ± 0.20^b***^
HbA1c (%)	5.76 ± 0.02^a^	5.78 ± 0.01^a^	5.69 ± 0.01^b^	5.67 ± 0.01^b***^
Insulin resistance (*N*, %)	466 (10.4)	1846 (11.7)^⁑^	485 (5.61)	1807 (6.07)
Serum total cholesterol (mg/dL)	193 ± 0.80^b^	192 ± 0.47^b^	199 ± 0.59^a^	200 ± 0.34^a***^
Serum HDL (mg/dL)	49.2 ± 0.29^b^	49.7 ± 0.17^b^	57.5 ± 0.21^a^	57.0 ± 0.12^a***#^
Serum LDL (mg/dL)	117 ± 0.73^b^	116 ± 0.43^b^	119 ± 0.55^a^	120 ± 0.31^a***^
Serum triglyceride (mg/dL)	136 ± 1.83^a^	133 ± 1.08^a^	113 ± 1.36^b^	116 ± 0.78^b***##^
SBP (mmHg)	126 ± 0.32^a^	126 ± 0.19^a^	120 ± 0.20^b^	121 ± 0.17^b***^
DBP (mmHg)	78.6 ± 0.21^a^	78.4 ± 0.13^a^	74.0 ± 0.16^b^	74.3 ± 0.10^b***^
Serum hs-CRP (mg/dL)	0.17 ± 0.01^a^	0.15 ± 0.01^a^	0.13 ± 0.01^b^	0.12 ± 0.004^b**+^
GFR (ml/min/1.73 m^2^)	81.9 ± 0.35^b^	82.4 ± 0.21^b^	87.4 ± 0.26^a^	87.7 ± 0.15^a***^
Serum AST (U/L)	25.1 ± 0.26^a^	25.3 ± 0.15^a^	22.7 ± 0.19^b^	22.8 ± 0.11^b***^
Serum ALT (U/L)	25.5 ± 0.39^a^	25.7 ± 0.23^a^	19.9 ± 0.29^b^	20.0 ± 0.16^b***^

Values represent means and standard deviations for continuous variables and numbers and percentages for categorical variables. The cutoff of Jang intake was 1.9 g/day. Covariates for adjustment included age, energy intake, residence area, education, income, sodium and alcohol intake, smoking status, and physical activity. BMI, body mass index, SMI, skeletal muscle index; HbA1c, hemoglobin A1c; HOMA-IR, Homeostatic Model Assessment for Insulin Resistance (cutoff: 2.32 of HOMA-IR); HDL, high-density lipoprotein; LDL, low-density lipoprotein; SBP, systolic blood pressure; DBP, diastolic blood pressure; hs-CRP, high-sensitive C-reactive protein; AST, aspartate aminotransferase; ALT, alanine aminotransferase. ^‡‡‡^Significantly different from the low-Jang group in *χ*^2^ test in each gender at *p* < 0.001. ^***^Significant differences by genders at *p* < 0.001. ^+^Significant differences by Jang intake at *p* < 0.05. ^#^Significant interaction between genders and Jang intake at *p* < 0.05 and ^##^ at *p* < 0.01. ^a,b,c,d^Different superscripts on the values indicated significant differences among the groups in Tukey’s test at *p* < 0.05.

**Figure 2:**
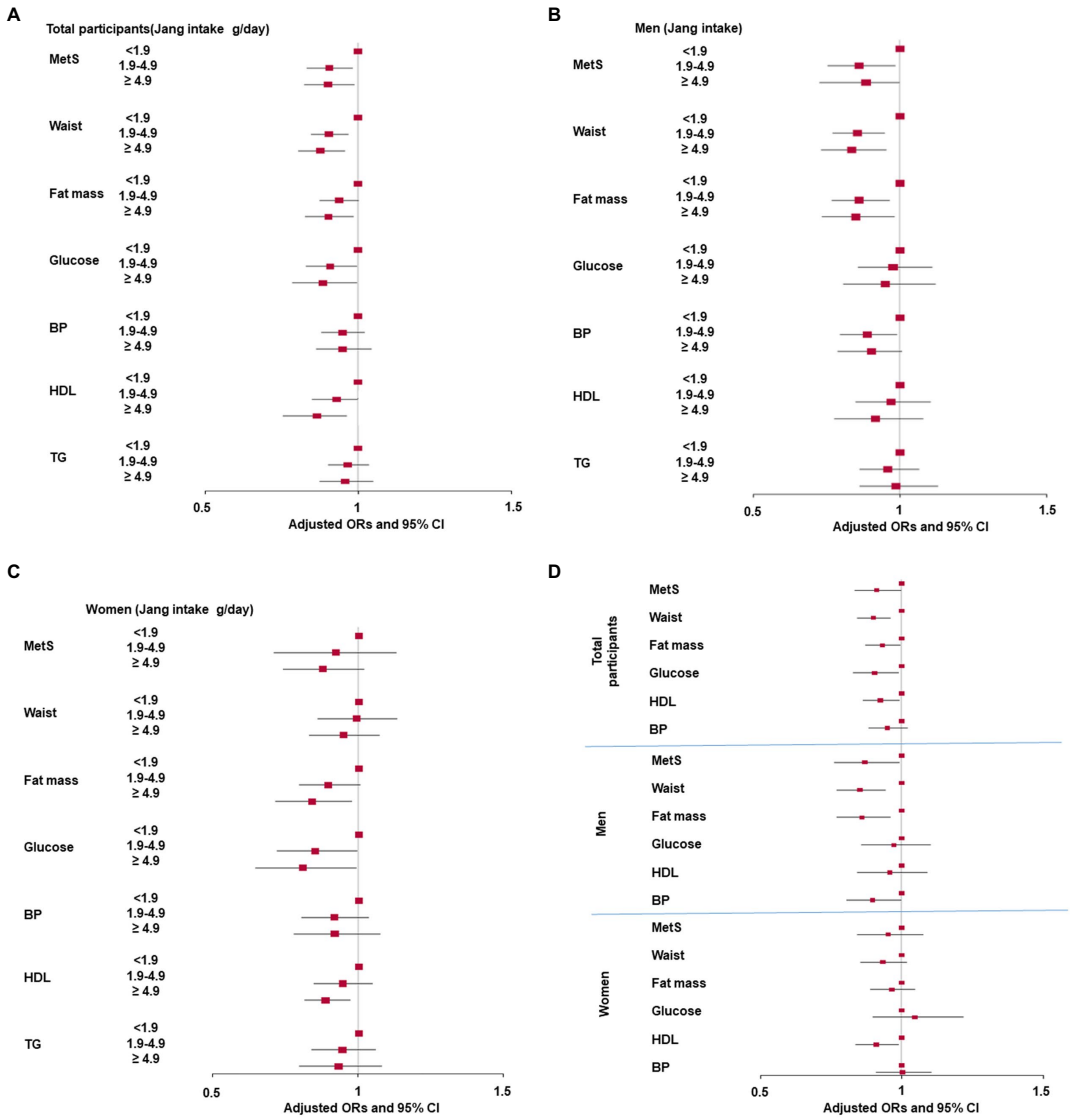
Association of Jang intake with metabolic syndrome (MetS) and its components according to gender **(A)** Adjusted odds ratio and 95% confidence intervals of MetS with Jang intake by tertiles in total participants **(B)** Adjusted odds ratio and 95% confidence intervals of MetS and its components with tertiles of Jang intake in men. Low intake <1.9 g Jang/day; Medium intake: 1.9 – <4.9 g/day; High intake: ≥4.9 g/day. **(C)** Adjusted odds ratio and 95% confidence intervals of MetS and its components with tertiles of Jang intake in women. Low intake <1.9g Jang/day; Medium intake: 1.9 – <4.9 g/day; High intake: ≥4.9 g/day, and **(D)** Adjusted odds ratio and 95% confidence intervals of MetS and its components with low-and high-Jang intake. (Cutoff: 1.9 g Jang intake/day) The cutoff points for logistic regression were as follows: MetS criteria; < 90 cm for men and 85 cm for women waist circumferences; <25% for men and 30% for women for fat mass; <110 mL/dL fasting serum glucose plus diabetic drug intake; <40 mg/dL for men and 50 mg/dL for women serum HDL cholesterol; <140 mmHg systolic blood pressure (SBP) or diastolic blood pressure (DBP) < 90 mmHg plus hypertension medication.

Jang, a fermented soybean containing salts, provides a salty and umami taste to food to enhance the flavors in Korean-style cooking. Overall, the Jang intake in tertiles was inversely associated with MetS risk and was inversely associated with specific components of MetS, such as waist circumference, fat mass, and serum glucose and hypo-HDL concentrations, after adjusting for age, energy intake, residence area, education, income, alcohol intake, smoking status, physical activity, and sodium intake. Figure 2A. The association between MetS and Jang intake was seen in gender differences. In the tertile classification of Jang intake, Jang intake was inversely associated with MetS only in men but not women (Figures 2B,C). In MetS components, Jang intake showed an inverse association with waist circumference, fat mass, and blood pressure in men (Figure 2B). However, in women, it was inversely associated with fat mass and serum glucose and hypo-HDL concentrations (Figure 2C).

When Jang intake was categorized into two groups, the high Jang intake was inversely associated with waist circumference, fat mass, and serum glucose and hypo-HDL concentrations, but not blood pressure, in all participants (Figure 2D). In men, Jang intake showed an inverse association with waist circumferences, fat mass, and blood pressure whereas, in women, Jang intake was inversely associated only with serum hypo-HDL concentrations (Figure 2D).

## Discussion

Hansik, a traditional Korean diet, is shown to be inversely associated with MetS risk (10). This nutritional benefit of Hansik is partly related to Jang intake. Jang is made of fermenting soybeans and salt and is used to promote the flavor and taste of Korean dishes and provide a salty taste. The complementary amino acids provided by soybean are essential nutrients for Asians who consume grains, especially rice, as their staple food. Jang has been extensively used as a salt substitute in cooking many Korean dishes. Jang intake results in a high salt intake. Due to high salt content, it has been suspected that Jang intake may also be associated with metabolic diseases. However, previous animal research has shown that when Jang substitutes salt in the diet, it is beneficial in preventing hypertension and obesity and managing menopausal symptoms (28). The present study demonstrated that high salt intake was positively associated with the risk of MetS and its components, namely waist circumference and fat mass, and inversely associated with serum HDL concentrations in both genders. However, interestingly, Jang intake was inversely associated with MetS and its components, especially waist circumference, body fat, hyperglycemia, and hypo-HDL cholesterolemia in all participants after adjusting for potential confounders, including sodium intake in this large city hospital-based cohort (*n* = 58,701). Their association was better in men than women. Jang intake was inversely associated with hypertension only in men and hypo-HDL cholesterolemia only in women. Therefore, MetS risk can be lowered when salt is replaced with Jang.

Excess salt intake is associated with an increased risk of hypertension, obesity, stroke, cardiovascular diseases, and stomach cancer (3, 4, 7). The common etiology of the diseases is increased insulin resistance and immunity, but their mechanisms are controversial. A high sodium intake (5 g/day) increases insulin resistance after adjusting for age and caloric intake in a randomized clinical trial of 160 obese Korean participants compared to a low sodium intake (2 g/day) (29). It may be related to several pathways: (1) High sodium increases serum leptin levels to decrease energy expenditure and increase energy intake, leading to hypertrophy of abdominal fat and making a vicious cycle (30), (2) High sodium elevates GLUT4 contents to increase glucose uptake in white adipose tissue, contributing to adipocyte hypertrophy (31), and (3) High sodium intake impairs glucose-stimulated insulin secretion by the attenuated β-cell mass increment to induce glucose intolerance (32). Furthermore, a high salt intake also modulates immunity by stimulating inflammatory macrophage and T cells, not neutrophils, and altering intestinal microbiota composition (33). It activates regulatory T cells (Tregs) and T helper (TH) 17 cells, which is possibly linked to promoting autoimmune diseases (34, 35).

Due to its high salt content, the health benefits of Jang have remained controversial. Since the olden days, Koreans have substituted salt with various types of Jang (fermented soybeans with salts) to enhance the flavor of foods. The present study has shown that higher sodium intake (≥3.31 g/day; 80th percentile) was positively associated with MetS risk in both genders. However, an equivalent sodium intake from Jang showed an inverse relationship with MetS risk. Isoflavonoids and peptides in Jang potentially protect against high sodium-induced insulin resistance and activation of Tregs and Th17 pathways (36). Therefore, excess sodium intake potentially increases MetS risk, but substituting salt with Jang may not lead to an increased MetS risk.

However, the mechanism by which regular consumption of Jang lowers the risk of MetS remains unknown. One proposed mechanism is related to aldosterone’s role in sodium and water retention. Salt-dependent hypertension is associated with volume expansion, renal dysfunction, impaired renin-angiotensin-aldosterone pathway, and central stimulation of the sympathetic nervous system activity. An earlier study has shown that the intake of Kanjang instead of salt (equivalent sodium intake) leads to lower serum aldosterone concentrations, thereby alleviating menopausal symptoms in estrogen-deficient mice (37). Kanjang intake also reduced visceral fat and serum glucose concentrations and elevated serum HDL concentrations in these mice (37). Similar results were seen in the present study wherein Jang intake was inversely associated with fat mass and serum glucose concentrations and positively associated with serum HDL concentrations in adults aged over 40 when they consumed an equivalent amount of sodium through Jang.

Potassium intake is also known to counteract the effects of sodium on body water retention. Potassium intake (adequate intake = 4,700 mg/day) needs to increase in proportion to sodium intake (adequate intake = 2000 mg/day) (38). However, in the present study, the dietary potassium-to-sodium ratio was lower in the high-Jang group (0.95 for men and 1.07 for women) than in the low-Jang group (1.13 for men and 1.27 for women) in both genders, and it was much lower than the recommended ratio (2.45). These results suggest that the inverse association of MetS risk with the high-Jang intake may not be related to the dietary potassium-and-sodium ratio. Dietary magnesium and calcium intakes are also associated with a lower MetS risk (39–41). In an animal study, sea salt intake decreased body fat in diet-induced obese animals compared to regular salt, which was associated with the high magnesium and sulfur content of sea salt (42). In a meta-analysis of six cross-sectional studies, the dietary magnesium intake exhibited a weighted inverse association with MetS risk. The overall MetS risk was lowered by 17% with every 100 mg/day increment of magnesium (43). Jang is made of soybean and sea salt, and a high Jang intake may increase magnesium consumption. The magnesium consumption was higher in the high-Jang intake group than in the low-Jang intake group in both genders in the present study. Therefore, magnesium could have contributed to the reduction in MetS risk. Similarly, it is well-known that dietary calcium intake lowers MetS risk (39, 40). Dietary calcium intake was inversely associated with the MetS risk in a meta-analysis involving 14,906 MetS patients. Increasing calcium intake by an additional 300 mg/day decreased MetS risk by 7% (44). In the present study, the adults in the high-Jang intake group had 33% higher dietary calcium levels than those in the low-Jang intake group. Therefore, the intake of both calcium and magnesium could lower the MetS risk in the high-Jang intake group compared to the low-Jang intake group.

Jang is made by fermenting soybeans containing salts but also includes abundant flavonoids and isoflavonoids. The primary isoflavonoids in soybean are daidzein, genistein, S-equol, and glycitein. Daidzein is converted to S-equol in the gut by specific bacteria after consuming fermented soybeans. S-equol is a potent phytoestrogen that can alleviate menopausal symptoms and MetS (45, 46). In human and animal studies (38, 39, 46, 47), isoflavonoid intake has been seen as beneficial for hypertension, dyslipidemia, and hyperglycemia (47, 48). In the present study, the isoflavonoid intake was 60–70% higher in the high-Jang intake group compared to the low-Jang group. The isoflavonoid intake from Jang has potentially influenced the improvement in MetS risk. Along with isoflavonoids, soy proteins (40 g/day) and peptides decrease blood pressure in patients with hypertension (49). Soybean peptides have an angiotensin-converting enzyme (ACE) and dipeptidyl-dipeptidases-IV inhibitory action, lowering hyperglycemia and blood pressure (50, 51). Therefore, Jang consumption, which contains isoflavonoids and soy peptides, could lower the risk of MetS and its components.

Soybeans, traditionally consumed in Asian countries, are usually fermented with *Bacillus* species and function as a synbiotic – providing the advantages of both probiotics and prebiotics. Miso intake, the Japanese soybean paste containing salt, has been reported to attenuate sympathovagal imbalance toward a more parasympathetic nerve dominant state and brain sodium sensitivity in mice administered a high-sodium solution injection (0.28 M sodium) and salt-sensitive rats given 1.3% salt solution (52, 53). Chungkookjang, Doenjang, Kanjang, and Kochujang intakes have been shown to alleviate hyperglycemia, dyslipidemia, obesity, and hypertension in earlier human and animal studies (6, 13–17). The *Bacillus* species in Chungkookjang, Doenjang, Kanjang, and Kochujang have been extensively studied, and certain isolated *Bacillus* species have probiotic characteristics*. Bacillus subtilis, Bacillus amyloliquefaciens,* and *Bacillus velezensis* have fibrinolytic activity, ACE inhibitory activity. and antioxidant activity (54–56). Therefore, fermented soybeans could act as synbiotics, and their intake can lower the risk of MetS and its components, including hypertension, dyslipidemia, obesity, and hyperglycemia.

This large cohort study was novel in establishing the benefits of fermented Jang on the components of MetS, particularly in men. However, this study has some limitations. First, the results could not show a cause-and-effect relationship due to using data from studies with a cross-sectional design. Second, the quantum of Jang intake was calculated by aggregating the consumption of Doenjang, Doenjang soup, Chungkookjang, and Ssamjang (the combination of Doenjang plus Kochujang) based on self-reporting by participants. The quantity reported by the participants could be over or underreported. Kanjang intake was excluded from the total Jang intake, which could have resulted in some bias in the Jang intake measurements. However, Kanjang and table salt were used as condiments to cook foods, and their intake was included in the SQFFQ. However, their usage might be varied in the same food as the individuals, and the variation could not be reflected. Therefore, Doenjang, Doenjang soup, Chungkookjang, and Ssamjang in Korean diets can be representative foods for Jang intake.

In conclusion, Jang, fermented salty soybeans traditionally consumed as part of the Korean diet, was found to be inversely associated with the risk of MetS and its components, including waist circumference, body fat, and hypertension in men in this large hospital-based cohort study. Moreover, Jang intake was inversely linked only to hypo-HDL cholesterolemia in women. Therefore, not only a decrease in sodium intake but also substituting salt for Jang in cooking may prevent and alleviate MetS risk by improving gut microbiota composition and providing isoflavonoids. The results can be applied to sodium intake in Asian countries where salt is used to promote flavor. These results need to be validated through a large and well-controlled randomized clinical trial or a longitudinal study.

## Data availability statement

The raw data supporting the conclusions of this article will be made available by the authors, without undue reservation.

## Ethics statement

The studies involving human participants were reviewed and approved by The Institutional Review Board of the National Institute of Health, Korea (KBP-2015-055) and Hoseo University, Korea (HR-034-01). The patients/participants provided their written informed consent to participate in this study.

## Author contributions

SP and DJ: conceptualization. H-JY, S-JJ, and MR: methodology. H-JY: resources. S-JJ and MR: data collection and analysis. SP: writing—original draft preparation. DJ and S-JJ: writing—review and editing. DJ, SP, and H-JY: supervision. All authors have read and agreed to the published version of the manuscript.

## Funding

This work was supported by “functional research of fermented soybean food (safety monitoring)” under the Ministry of Agriculture, Food and Rural Affairs and partly Korea Agro-Fisheries and Food trade corporation.

## Conflict of interest

The authors declare that the research was conducted in the absence of any commercial or financial relationships that could be construed as a potential conflict of interest.

## Publisher’s note

All claims expressed in this article are solely those of the authors and do not necessarily represent those of their affiliated organizations, or those of the publisher, the editors and the reviewers. Any product that may be evaluated in this article, or claim that may be made by its manufacturer, is not guaranteed or endorsed by the publisher.
